# Ausgeprägter Wessely-Immunring bei Keratitis – ein Chamäleon

**DOI:** 10.1007/s00347-020-01084-8

**Published:** 2020-03-24

**Authors:** Isabel Weinstein, Fabian N. Fries, Nóra Szentmáry, Berthold Seitz, Loay Daas

**Affiliations:** Klinik für Augenheilkunde, Universitätsklinikum des Saarlandes UKS, Kirrberger Str. 100, 66421 Homburg, Deutschland

**Keywords:** Ringförmige Hornhauttrübung, Properdin-Ring, Akanthamöbenkeratitis, *Pseudomonas aeruginosa*, Kontaktlinsenassoziierte Keratitis, Ring-shaped corneal opacity, Properdin-mediated immune ring, Acanthamoeba keratitis, *Pseudomonas aeruginosa*, Contact lens-associated keratitis

## Abstract

Ein Wessely-Immunring kann bei verschiedenen kornealen Infektionen sowie bei nichtinfektiöser Ätiologie auftreten und differenzialdiagnostisch wegweisend, aber auch irreführend sein. Eine definitive Diagnosestellung kann nur in Gesamtschau der klinischen und mikrobiologischen Befunde erfolgen. Differenzialdiagnostische Überlegungen und Therapiestrategien werden im Kontext der Kasuistik eines 31 Jahre alten Kontaktlinsenträgers mit diffuser Endotheldekompensation bei fokalem mittelperipherem Infiltrat mit Wessely-Immunring exemplarisch erörtert und kritisch reflektiert.

## Abstract (en)

A Wessely immune ring can be found in various corneal infections as well as in non-infectious processes. Its appearance can aid in the differential diagnosis but can also be misleading. A definitive diagnosis can only be reached when all clinical and microbiological findings are taken into consideration. This article discusses and critically reflects on the differential diagnostic considerations and treatment strategies in the context of a case report on a 31-year-old patient with contact lenses and diffuse endothelial decompensation with a focal, mid-peripheral infiltrate and a Wessely immune ring.

## Anamnese

Ein 31-jähriger Kontaktlinsenträger wurde notfallmäßig mit der Verdachtsdiagnose einer kontaktlinsenassoziierten Akanthamöbenkeratitis und zunehmender Befundverschlechterung von seinem Hausaugenarzt an unsere Klinik überwiesen. Die Beschwerden bestanden zu dem Zeitpunkt seit 3 Tagen und wurden mit Ciprofloxacin Augentropfen (AT) stündlich sowie Ofloxacin Augensalbe (AS) zur Nacht behandelt. Bei stationärer Aufnahme beklagte der Patient v. a. eine Photophobie, eine Rötung, wenige Schmerzen sowie einen Visusabfall am betroffenen rechten Auge. In der Augenanamnese konnte eine Myopia permagna eruiert werden, Allgemeinerkrankungen sowie eine Familienanamnese bezüglich ophthalmologischer Erkrankungen waren leer.

## Klinischer Befund

**Figure 1:**
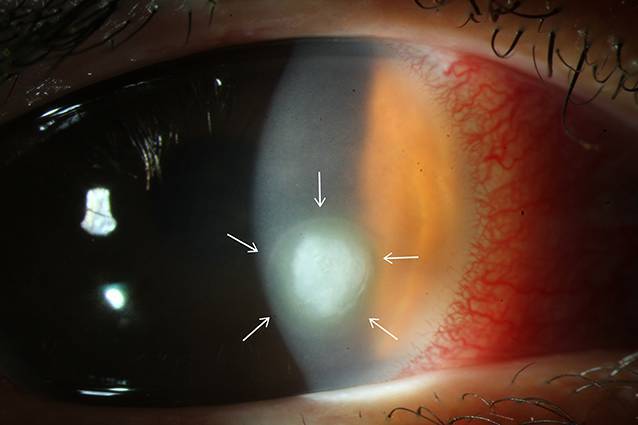

In der Spaltlampenmikroskopie imponierten am betroffenen rechten Auge eine ausgeprägte gemischte konjunktivale Injektion sowie eine diffuse Endotheldekompensation bei fokalem mittelperipherem Infiltrat mit ausgeprägtem Wessely-Immunring (Abb. 1). Die Vorderkammer war tief und reizfrei, die Linse zeigte sich altersentsprechend klar. Ein Funduseinblick am betroffenen rechten Auge war zum Zeitpunkt der stationären Aufnahme aufgrund der Hornhautdekompensation nicht möglich. Sonographisch konnten aber ein reizfreier Glaskörper sowie eine zirkuläre Netzhautanlage gesichert werden. Der bestkorrigierte Visus rechts betrug 0,16. Am Partnerauge zeigte sich ein altersentsprechender Normalbefund mit bestkorrigiertem Visus von 1,0. Der Augeninnendruck lag applanatorisch gemessen beidseits bei 12 mm Hg.

## Diagnose

Bei klinischem Verdacht auf eine Akanthamöbenkeratitis wurde der Patient lokal mit Polyhexanid, Propamidinisoethionat und Neomycin AT sowie, um eine mykotische Mischinfektion abzudecken, systemisch mit Voriconazol behandelt. Konfokalmikroskopisch konnten weder Akanthamöbenzysten noch Pilzhyphen nachgewiesen werden, und auch die PCR auf Akanthamöben erwies sich als negativ. Im Hornhautabstrich lieferte die mikrobiologische Kultur nach 5 Tagen einen Nachweis von *Pseudomonas aeruginosa* in hoher Anzahl.

## Therapie und Verlauf

**Figure 2:**
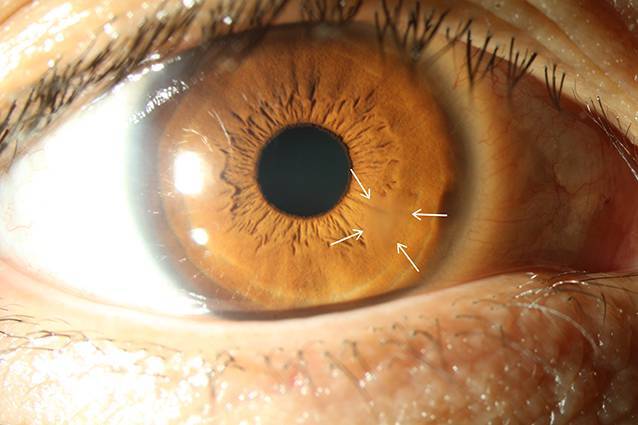

Es erfolgte eine Therapieumstellung auf Tobramycin 5 %, Cefuroxim 5 % und Polyhexanid AT sowie Ceftriaxon systemisch. Hierunter kam es rasch zu einer Befundbesserung, einem Rückgang des Wessely-Immunrings nach 5 Tagen und einer narbigen Ausheilung (Abb. 2) der Keratitis sowie zu einem Visusanstieg von initial 0,16 auf 1,0 bei Entlassung.

## Diskussion

Ein Wessely-Immunring stellt einen unspezifischen Leitbefund dar, welcher bei verschiedenen Keratitiden infektiöser sowie nichtinfektiöser Genese auftreten kann [6]. Er kann hierbei differenzialdiagnostisch wegweisend, aber auch irreführend sein. Eine definitive Diagnosestellung kann nur in Gesamtschau der klinischen und mikrobiologischen Befunde erfolgen.

Der Wessely-Immunring entsteht durch eine entzündliche Zellinfiltration aus Antigen-Antikörper-Komplexen im Hornhautstroma. Antigene diffundieren radiär in Richtung Hornhautperipherie und treffen dabei auf Antikörper, welche vom Limbus ausgehend in die Kornea wandern. Dieser Antigen-Antikörper-Komplex veranlasst polymorphkernige Entzündungszellen zur Migration und führt folglich zur Ausbildung der ringförmigen Hornhauttrübung [1, 2]. Da die Geschwindigkeit der Antigendiffusion vom Pathogen Richtung Peripherie in alle Richtungen ähnlich und entscheidend geringer als die aktive Migration der Entzündungszellen vom Limbus ist, entsteht typischerweise ein nach außen homogener Ring, während das Pathogen sich in dessen Zentrum befindet (Abb. 1). Dabei kann der Wessely-Immunring komplett oder auch inkomplett ausgebildet sein.

Ein Wessely-Immunring zeigt sich oft erst im fortgeschrittenen klinischen Verlauf als Spätbefund, im Falle einer Akanthamöbenkeratitis typischerweise nach 10 bis 14 Tagen [1, 2, 8]. Hierbei ist das Hornhautareal in der Mitte des Immunringes bei Akanthamöbenkeratitiden häufig klar, während sich bei bakteriellen oder mykotischen Koinfektionen ein Hornhautinfiltrat im Zentrum des Immunringes zeigt.

Allerdings kann der Immunring auch durch eine alternative Komplementaktivierung als Properdin-Ring Antikörper-unabhängig bereits nach 1 bis 2 Tagen auftreten.

Properdin ist ein Gammaglobulin, welches eine Schlüsselrolle in der positiven Regulierung des alternativen Weges der Komplementkaskade in der Immunabwehr spielt und die Phagozytose beeinflusst. Properdin bindet die C3-Konvertase der alternativen Komplementaktivierung, welche u. a. durch bakterielle Endotoxine und somit Antikörper-unabhängig aktiviert wird.

Diese Properdin-vermittelten Immunringe werden v. a. bei gramnegativen Bakterien, besonders bei *Pseudomonas aeruginosa*, beschrieben [2, 4, 5].

Weitere Aktivatoren des alternativen Komplementweges stellen IgA-Immunkomplexe, Polysaccharide und Zellwandbestandteile dar.

**Figure 3:**
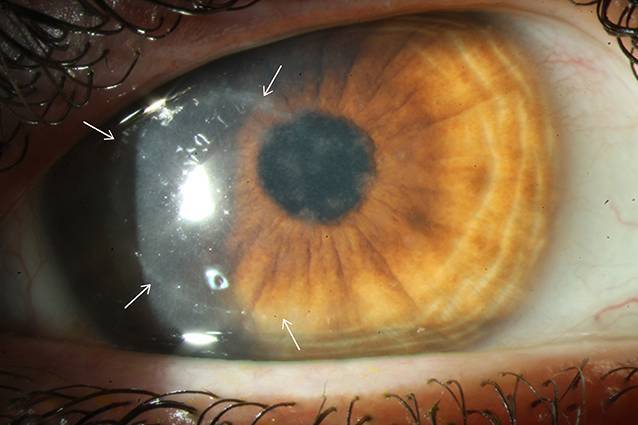

**Figure 4:**
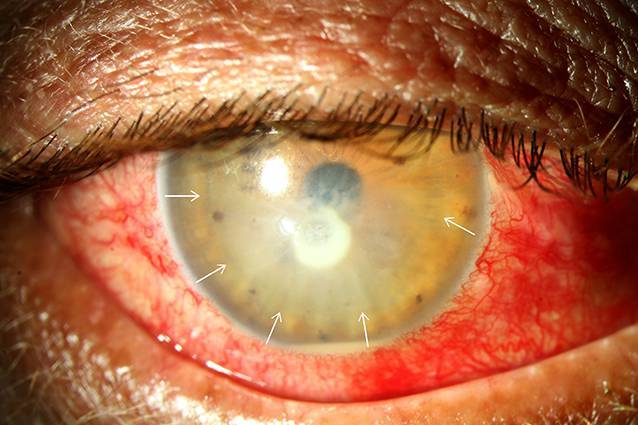

**Figure 5:**
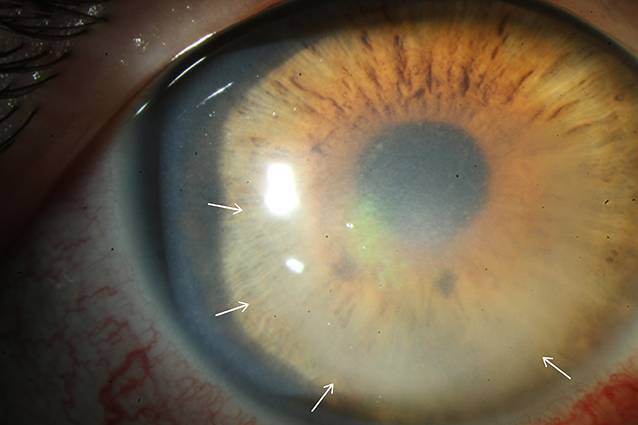

Differenzialdiagnostisch sollten bei Vorliegen eines Wessely-Immunrings neben einer Akanthamöbenkeratitis (Abb. 3) auch eine mykotische Keratitis (Abb. 4), Keratitiden bakterieller Genese sowie im Entferntesten eine Herpes-simplex-Keratitis (Abb. 5) in Betracht gezogen werden.

Des Weiteren können Wessely-Immunringe ebenfalls aufgrund nichtinfektiöser Genese im Rahmen rezidivierender Erosiones [3], Abusus von Lokalanästhetika [2], nach Verätzung, nach Kontakt mit antigenen Hornhautfremdkörpern oder als Folge kornealer Insektenstiche beobachtet werden [2, 8].

Die Ätiologie der Entstehung der nichtinfektiösen Immunringe ist hierbei nicht vollständig geklärt. Es werden sowohl die klassische Antigen-Antikörper-Komplex-Aktivierung, die alternative Komplementaktivierung sowie weitere Wege wie Kreuzreaktionen von bakteriellen und humanen Heat-Shock-Proteinen diskutiert [7].

## Fazit für die Praxis

- Ein Wessely-Immunring kann für die Diagnosestellung wegweisend, aber auch irreführend sein.
- Differenzialdiagnostisch sollten bei Vorliegen eines Wessely-Immunrings neben einer Akanthamöbenkeratitis auch eine mykotische Keratitis, Keratitiden bakterieller Genese sowie im Entferntesten eine Herpes-simplex-Keratitis in Betracht gezogen werden.
- Er tritt bei Akanthamöbenkeratitiden typischerweise nach 10 bis 14 Tagen auf, kann aber bereits nach 1 bis 2 Tagen Properdin-vermittelt auftreten, hier v. a. bei *Pseudomonas aeruginosa*.
- Eine definitive Diagnosestellung kann nur in Zusammenschau aller klinischen und mikrobiologischen Befunde erfolgen.
